# Increasing coverage of insecticide-treated nets in rural Nigeria: implications of consumer knowledge, preferences and expenditures for malaria prevention

**DOI:** 10.1186/1475-2875-4-29

**Published:** 2005-07-18

**Authors:** Obinna Onwujekwe, Benjamin Uzochukwu, Nkoli Ezumah, Elvis Shu

**Affiliations:** 1 Health Policy Research Unit, Department of Health Administration and Management, College of Medicine, University of Nigeria Enugu-Campus, Nigeria; 2 Department of Community Medicine, College of Medicine, University of Nigeria Enugu-Campus, Nigeria; 3 Department of Sociology and Anthropology, University of Nigeria, Nsukka, Nigeria; 4 Department of Pharmacology and Therapeutics, College of Medicine, University of Nigeria Enugu-Campus, Nigeria

## Abstract

**Background:**

The coverage of insecticide-treated nets (ITNs) remains low despite existing distribution strategies, hence, it was important to assess consumers' preferences for distribution of ITNs, as well as their perceptions and expenditures for malaria prevention and to examine the implications for scaling-up ITNs in rural Nigeria.

**Methods:**

Nine focus group discussions (FGDs) and questionnaires to 798 respondents from three malaria hyper-endemic villages from Enugu state, south-east Nigeria were the study tools.

**Results:**

There was a broad spectrum of malaria preventive tools being used by people. The average monthly expenditure on malaria prevention per household was 55.55 Naira ($0.4). More than 80% of the respondent had never purchased any form of untreated mosquito net. People mostly preferred centralized community-based sales of the ITNS, with instalment payments.

**Conclusion:**

People were knowledgeable about malaria and the beneficial effects of using nets to protect themselves from the disease. The mostly preferred community-based distribution of ITNs implies that the strategy is a potential untapped additional channel for scaling-up ITNs in Nigeria and possibly other parts of sub-Saharan Africa.

## Introduction

A current challenge that is facing many sub-Saharan African countries like Nigeria is how to achieve widespread distribution and use of insecticide-treated nets (ITNs) for the control of malaria. The Africa Malaria Report shows that many countries are quite far from reaching the target of 60% ITNs coverage in sub-Saharan African countries by the year 2005, which was set in Abuja by the African Heads of State for the provision of ITNs to children under five and to pregnant women [1]. Malaria is the number one public health problem in Nigeria [2,3]. By preventing malaria, ITNs reduce the need for treatment and the pressure on health services [2,3]. ITNs were recently added as a malaria control policy in Nigeria and the government wishes to scale-up the use of ITNs.

The determination of distribution mechanisms that will assure high coverage with the ITNs, especially in rural areas, remains a topical issue in Nigeria and in many sub-Saharan African countries (SSA). The public health care system was initially used to distribute ITNs in Nigeria, but the coverage was quite low. Currently, commercial sector distribution and social marketing of ITNs is being promoted in some states in Nigeria, but the coverage remains low. Community-based distribution has not been tried on a large scale, but it could possibly be added to existing strategies to successfully distribute and scale-up ITNs in rural areas. Similar strategies are being successfully used for the distribution of ivermectin for the control of onchocerciasis [4], filters for the control of guinea-worm [5] and home management of malaria [6].

Chavasse et al [7] identified four 'most common options' for ITN and insecticide distribution and these were: government systems; non-governmental organization systems; unassisted private sector; and assisted private sector (social marketing models). The current major strategies for scaling-up of ITNs in SSA are social marketing and use of the commercial sector [3,8-11]. However, it has been argued that the ITNs distribution approach chosen must depend primarily on local circumstances, as experience gives no clear general reason to prefer one option over another [8,12].

This paper examines the perceptions, expenditures and preferences of consumers for the prevention of malaria, as well as ITNs ownership and preferred strategies for distributing ITNs. Such information should precede the design of sustainable and effective locally relevant strategies for scaled-up distribution of ITNs. The information will also inform policy makers on the nature of information, education and communication campaigns that could be used to increase ITNs coverage. A successful social marketing strategy for ITNs in Tanzania was developed based on a similar assessment [13]. The information could also reveal the existing and potential demand for mosquito nets and provide channels for the optimal strategies for financing the ITNs.

## Methods

### Study area

The study was conducted in three villages in Achi community, Oji-river local government area (LGA) of Enugu State, south-east Nigeria. The villages were Ahani, Amaetiti and Enugwu-Akwu. Achi has a high malaria transmission rate, year round, with an average malaria incidence rate of 15%[14]. The major malaria vector in Achi is *Anopheles gambiae*, while *Plasmodium falciparum *causes more than 90% of all malaria infections. Achi has an estimated population of 45,000 people. Untreated mosquito nets and ITNs are not sold in these villages, but they are sold in urban areas like Enugu and Onitsha. It usually takes about one hour to reach Enugu and one hour and 20 minutes to reach Onitsha by bus.

### Data collection

Qualitative data was collected through three focus group discussions (FGDs), with separate groups of men, women and youths. Hence, a total of three FGDs were held in each village (total of nine). The number of participants per FGD ranged from six to nine people and the participants were purposively selected with the help of the village heads, so that all sections of the villages were represented. The agegroups for men and women were from 35 to 60 years and for youths 20 to 34 years. Youth groups had equal numbers of male and female participants. A discussion guide was used to direct the discussions during the FGDs, which were moderated by a social scientist. Each lasted a maximum of two hours. The discussions focused on causes of malaria, how to prevent the disease, level of net ownership, preferences for distributing and paying for the nets within their villages.

Quantitative data was collected using a pre-tested interviewer-administered questionnaire that was administered to respondents from a total of 900 households (300 from each village). Adequate sample size was determined, using a power of 80%, 95% confidence level and a malaria incidence rate of 15%. The EPI-info software programme was used to calculate the sample size. The heads of households or their representatives (if the household head was not available) from the selected households were interviewed. The participants to the FGDs were excluded from the quantitative survey so as not to bias the results, since they already had better information than others. Data was collected on the socio-economic and demographic characteristics of the households, the level of their ownership of mosquito nets and their expenditure on the prevention of mosquito nuisance and malaria in the month prior to the survey. Expenditure included mosquito nets, coils, insecticide-sprays, drugs, body creams etc. The Ethics Committee of the University of Nigeria Teaching Hospital, Enugu, Nigeria approved the study.

### Data analysis

The variables that were explored by both the FGDs and the questionnaire focused on determining the most common diseases in the community, local names for malaria, the different types of malaria, its symptoms and causes. Other points explored were the perception of mosquito nuisance, the subject' s health care seeking behaviour, mosquito control effort and solution to the malaria problem. Participants were also asked their preferences for paying and distributing ITNs. The possible major avenues for delivery of ITNs, such as through the commercial sector, the public health system, community-based distribution and social marketing were explored. The records of the FGDs were transcribed on the same day the FGDs were held and content analysis was used to categorize the responses into domains representing the common themes. The areas of consensus and divergence in the responses according to the groups and villages were determined for better identification of factors that influence health seeking behaviour. Tabulations and tests of statistically significant differences using non-parametric chi-squared tests were used to analyse the quantitative data.

## Results

### Qualitative data

#### Causes and prevention of malaria

The local terminology for presumptive malaria was "Iba", which denotes fever. Many causes of Iba were identified by people, though as a consensus, most of the participants in all the groups identified mosquitoes as its major cause. The methods given by the participants on mosquito nuisance control included insecticide sprays, keeping doors and windows closed in the night, use of electric fans, applying mosquito repellent cream on the exposed body at night, clearing weeds around the house, wearing long dresses, applying kerosene to the body and in the house, use of mosquito nets and physically killing mosquitoes. Other mosquito control techniques were draining stagnant water and covering water containers, especially during the rainy season, and burning or placing local leaves (osigbu) in and around the house. The leaves are good mosquito repellents because of their smell. Some participants said that preventive drugs such as paludrine were used, especially by pregnant women.

#### Mosquito net ownership

The discussions showed that there was low mosquito net ownership and use in these three villages. A few Amaetiti youths said that they had used bed-nets in secondary school boarding houses. All the Enugu-Akwu women said that they did not use mosquito nets due to the high cost, while one of them said that nursing mothers used nets to cover their babies. Though, window-nets were preferred to bed-nets, lack of money was the major constraints on net ownership, as people indicated that they would have bought nets, but for the high cost. Window-nets were preferred over bed-nets because they provided better aeration to the users and did not generate heat. A woman from Amaetiti said, *"I will never use mosquito bed-nets even given free of charge. They make me feel as if I am being suffocated"*. Only one man from Enugu-Akwu had heard about ITNs through the radio. Many questioned their safety, since they felt that sleeping under nets treated with insecticides may be harmful. Most people were excited when told about ITNs and their mode of action and indicated their willingness to buy the nets when available, if affordable and not harmful.

#### Preferred distribution mechanisms for ITNs

Almost all groups preferred community-based distribution of the ITNs to sales by the commercial sector (e.g. patent medicine dealers), public health system and by health teams that occasionally visited the villages. The major reason for their preference was that community-based distribution would make access to the ITNs easier and the nets would not be too expensive, since the distributors would be resident in the villages and the nets would be sold at uniform prices that would be fixed by the government or the major suppliers. The majority of people felt that the profit motive could make the nets from the commercial sector very expensive. Also, they felt that teams coming to their villages to promote and sell the nets might come irregularly and, when they did, people might not have money to buy the nets. Most of the participants agreed that ITNs should be sold centrally within their communities. Some people also suggested the use of the door-to-door sales method for the nets, especially for people unable to go to the central location to buy the nets. However, the Ahani youths were against door-to-door sales. As one of them explained *"If the ITNs are taken from house to house, people will value them less, because they will feel that the government was unable to sell them and is begging them to buy the nets".*

#### Preferred mode of payments and pricing for ITNs

There was a group concensus that single full payment was the best payment system, if people could afford to pay the full price at once. However, in the event that the price of the nets is high, then instalment payments should be allowed. A one-off pre-payment system was not preferred, because income in rural areas fluctuates and even people in the formal wage-earning sectors, who get paid monthly, have competing needs, or their salaries may go unpaid for many months. Additionally, a one-off payment could prove to be a big burden, especially if a household purchases many nets. Some people from all the groups suggested that the nets should have a subsidized, but fixed price. In the words of a man from Amaetiti: *"The price of the nets should be very low. They should be fixed prices and the ward leaders will tell all households about the price"*. Another stated that *"Trained field workers should educate the people on the importance of the nets, and then seek their opinion on the prices for the net. Then, the average price should be used as what people should pay".*

### Quantitative data

The usable number of questionnaires for the analysis were 798. The respondents were mostly heads of households, middle-aged, females, married and farmers (Table 1). More than 87.1% of the respondents correctly identified mosquitoes as the cause of malaria, while 4.6% of them disagreed that mosquitoes cause malaria and the rest did not know whether they did (Table 2). A total of 76.2% of the respondents concurred that nets could prevent mosquito bites (this was for all kinds of nets), while 15.5%did not and the rest did not know. Though 71.4% of the respondents believed that insecticides could prevent mosquito bites, 20.9% did not believe that it could and the rest were not sure. About 83% of the respondents perceived that either they or other members of their household could contract malaria, while the rest did not. Chi-squared tests showed that all the differences in proportions were statistically significantly different (p < 0.0001).

**Table 1:** Socio-economic and demographic data of the respondents

Variables	N = 798 n (%)
Head of household	528 (65.2%)
Number of household residents	
Mean (Standard deviation)	4.62 (2.69)
The age of the respondent in years	
Mean (Standard deviation)	48.77 (15.55)
Male respondents	369 (46.2%)
Years of formal education	
Mean (standard deviation)	4.19 (4.76)
Whether the respondent ever married	703 (88.1%)
Occupation:	
• Unemployed/unskilled labourers/housewives	11 (1.4%)
• Farmers	475 (59.5%)
• Skilled labourers/petty traders/pensioners	215 (29.6%)
• Formally employed/regular wage earners/medium business people	62 (7.8%)
• Professionals/big business people	33 (4.1%)

**Table 2:** Knowledge respondents had about the cause and preventive measures for malaria

Variables	Yes n (%)	No n (%)	Do not know n (%)
Mosquitoes could cause malaria	695 (87.1%)	37 (4.6%)	66 (8.3%)
Nets could be used to prevent mosquito bites	608 (76.2%)	124 (15.5%)	66 (8.3%)
Insecticides could prevent mosquito bites	570 (71.4%)	167 (20.9%)	61 (7.6%)
Know where to buy a mosquito net	260 (32.6%)	538 (67.4%)	0
Know the current price of mosquito nets	46 (5.8%)	752 (94.2%)	0
Have ever heard of ITNs	91 (11.4%)	707 (88.6%)	0

n = 798

A small proportion (25.2%) of the households spent money on malaria prevention and the average monthly expenditure to prevent malaria was 55.55 Naira ($0.5) (Table 3). Conversely, the average monthly expenditure that people spent to treat malaria was 365.9 Naira ($3.05) with a 95% confidence interval of 306.8 Naira ($2.56) to 429.9 Naira ($3.58). People spent money on many preventive tools, including insecticide sprays, mosquito coils, window-nets, untreated bed-nets and drugs for chemoprophylaxis. More than 80% of the respondents had never purchased any form of untreated mosquito net. The nets that a few of the people had were window-nets. Only 32.6% claimed to know where to buy an untreated net, that is mainly the markets in Enugu and Onitsha. Just 5.8% of the respondents knew the current prices of untreated nets. There was very little prior knowledge about ITNs, as only 11.4% of respondents had ever heard of ITNs before the study. Nobody had ever purchased or re/treated an ITN in the three groups.

**Table 3:** Household expenditure to prevent malaria and level of acquisition of untreated nets

Variables	Values n = 798 n (%)
Households that had bought untreated nets before the survey	122 (15.3%)
Number of households that actually spent money to prevent malaria	202 (25.2%)
Amount households spent the previous month to prevent malaria (Naira)	
Mean (S.D.)	55.55 (175.6)
95% Confidence interval	43.45 – 67.84
Median	0.00

Note: • Chi-square of differences in numbers of people that bought untreated nets and those who did not = 198.0 (p < 0.0001)

• Chi-square of differences in numbers of households that spent money to prvent malaria and those who did not = 386.4 (p < 0.0001)

## Discussion

People were knowledgeable about the mode of transmission of malaria and the benefits of using malaria preventive methods such as nets and insecticides, but very few households spent money on malaria preventive tools. The acquisition and usage of untreated mosquito nets was low and nil for ITNs and very few people had heard about ITNs. The results of this study are comparable to those of a marketing survey in Nigeria, where 10% of 5,000 households owned at least one net [15]. The results are also similar to findings in Mozambique, where only 3% of people had heard about ITNs and 9% used treated or ordinary nets [16]. The use of untreated bed-nets, though uncommon in households, could be found in secondary school boarding houses used by students.

The lack of substantial expenditure on malaria prevention means that getting people to pay for ITNs at the unit cost of 450 Naira ($3.8) per net, which is the average prevailing market price of ITNs in Nigeria, would be an uphill task. People would need to be convinced to increase their budget on prevention so as to cover the expense of using nets, especially in areas without a net usage culture. This depends, too, on whether they perceive ITNs as a complement to or a substitute for existing malaria preventive measures. Since, health care expenditures are the minimum amount or lower bound estimate of the amount that people are willing to pay for health care [17], the expenditures on malaria prevention could be taken as the lower bound the respondents would pay for ITNs.

An implication of these findings for scaling up of ITNs in rural areas is that malaria control programme managers should design and fine-tune how community-based distribution of ITNs could be added to existing distribution strategies so that ITNs could penetrate into the rural areas in large numbers. Community-based distribution in this context involves the recruitment and training of community residents to become ITNs community-based distributors (CBDs). The CBDs could be recruited in conjunction with the community leaders and trained by promoters of ITNs distribution such as the state and local government malaria control programme officers as well as other non-governmental promoters of ITNs. It is hoped that the CBDs would regularly obtain the ITNs from both public and non-governmental sources for sale to their community members. For sustainability of community-based distribution, the CBDs should either be paid a stipend directly by the body that supplies them the nets or allowed to slightly add a mark-up on the sale prices of the nets, which they would collect as their commission. The primary healthcare system would be expected to supervise, monitor and evaluate the CBDs as well as provide them with continuous re-training. They will also train new CBDs, when there is CBD attrition.

The malaria control programme managers should also consider how they would tackle the villagers' reluctance to prefer the commercial sector and vertical teams, so that these other strategies would also be useful in rural areas in the medium to long term basis. This is because the community-based distribution strategy would be used alongside the distribution of the ITNs through public and private healthcare facilities as well as through the commercial sector.

The payment mechanism within community-based distribution of ITNs should be designed to limit the occurrence of payment defaulters. The mostly preferred instalment payment before ITN acquisition ensures that people will get the ITNs whenever available, even if they have irregular availability of cash. Nonetheless, a mixture of instalment payment and one-off payment could be used in order to avoid the pitfalls of instalment payment, where people default in completing their payments after collecting the ITNs. The village heads could be made to act as guarantors for people who would be allowed to pay by instalment. However, subsidy mechanisms such as vouchers [9], subsidies and exemptions could be used to financially protect the poor and high risk groups.

A cost-effective use of resources is the promotion of multiple compatible distribution strategies in communities by government and other organizations, so that there would be an appreciable scaling-up of the supply and use of ITNs. The use of multiple distribution strategies within rural areas is in line with the pluralistic approach which has been advocated by the WHO Strategic Framework for Scaling-Up ITNs [18]. Using single distribution methods such as social marketing or the commercial sector alone might not lead to widespread supply and use of ITNs, especially if the consumers do not really prefer such strategies.

What is needed to scale up ITNs in rural areas is more effort to develop and implement consumer preferred distribution strategies, which will complement more widely used distribution strategies such as the commercial sector and social marketing. Motivational health education would encourage people to increase their current low level of expenditure on malaria prevention, so that they would be able to buy ITNs and re-treatment services. Examples of where community-based distribution did not work [19] and where it worked [20] should guide the development and implementation of community-based distribution of ITNs.

## Authors' contributions

OO conceived and designed the study. All the authors participated in data collection and analysis. OO wrote the first draft and all the authors revised the drafts until the final draft was produced for publication.
